# Effects of Tactile Sensory Stimulation Training of the Trunk and Sole on Standing Balance Ability in Older Adults: A Randomized Controlled Trial

**DOI:** 10.3390/jfmk10010096

**Published:** 2025-03-17

**Authors:** Toshiaki Tanaka, Yusuke Maeda, Takahiro Miura

**Affiliations:** 1 Research Center for Advanced Science and Technology (RCAST), The University of Tokyo, Tokyo 113-8656, Japan; 2 Department of Physical Therapy, School of Health Sciences at Odawara, International University of Health and Welfare, Odawara 250-8588, Japan; y.maeda@iuhw.ac.jp; 3 National Institute of Advanced Industrial Science and Technology (AIST), Kashiwa 277-0882, Japan; miura-t@aist.go.jp

**Keywords:** dynamic standing balance, sensory stimulation training, older adults

## Abstract

**Background:** Aging is associated with a decline in both motor and sensory functions that destabilizes posture, increasing the risk of falls. Dynamic standing balance is strongly linked to fall risk in older adults. Sensory information from the soles and trunk is essential for balance control. Few studies have demonstrated the efficacy of targeted sensory training on balance improvement. **Objectives:** To assess vibratory sensation function in the trunk and sole using a vibration device and evaluate the effects of trunk and sole tactile sensation training on dynamic standing balance performance in older adults. **Methods:** In this randomized controlled trial, eighteen older adults were randomly assigned to three groups: control (n = 8, mean age 66.6 ± 3.4), trunk training (n = 5, mean age 71.0 ± 1.9), and sole training (n = 5, mean age 66.4 ± 3.6). The training lasted for 10 weeks, utilizing vibratory stimulation at 128 Hz through tuning forks for 15 min during each session, conducted three times a week. The primary outcomes were vibratory sensitivity, assessed with a belt-fitted device on the trunk and a plate equipped with vibrators on the soles, and dynamic balance, evaluated through force plate testing that measured limits of stability (LoS) in multiple directions. **Results:** Correct response rates for trunk vibratory stimulation significantly improved in the trunk training group (*p* < 0.05). The rate of two-stimuli discrimination improved in both training groups. Significant advancements in balance metrics were observed in the trunk and sole training groups when compared to the control group, especially regarding anterior–posterior tilts (*p* < 0.05). A positive correlation was identified between two-point vibratory discrimination and LoS test performance. **Conclusions:** Sensory training of the trunk and sole enhances balance performance in older adults, suggesting potential benefits for fall prevention. Future studies should assess long-term effects and explore optimal training duration with larger sample sizes.

## 1. Introduction

Aging is associated with a decline in both motor and sensory functions which destabilize posture, resulting in increased risk of injury, hospitalization, and even mortality due to falls [1]. One study reported that one-third of all older adults in the United States suffer falls every year [2], while the rate of falls among older adults in Japan every year is reported to be over 20% [3]. Fractures due to falls are a particularly major risk factor for older individuals to need care, while the gait and balance disorders accompanying a decline in muscle strength are a risk factor for falls [1].

The most frequently used technique to evaluate the ability of static and dynamic postural stability is the measurement of the position and displacement of the center of pressure (COP) by using a force plate as a clinical assessment [4,5]. Static balance is the ability to maintain postural stability and orientation with the center of mass over the base of support and the body at rest. Dynamic balance is the ability to maintain postural stability and orientation with the center of mass over the base of support while the body parts are in motion [6]. Moreover, dynamic standing balance is deeply related to falls in older adults [7,8]. Regarding dynamic balance, the maximum ability to move forward, backward, leftward, and rightward while standing was used as an important evaluation of dynamic balance ability [9].

Obtaining accurate sensory information from the soles of the feet is essential for the control of standing balance [10]. In our prior study, we developed a device to evaluate vibratory sensation as a form of tactile sensation [11]. Using this device, we showed that sensory function in the toes and the soles is lower in older people than in young people, and that sensation in the soles of the feet affects standing balance [11,12]. Other studies have reported that this tactile sensation of the legs is related to standing balance performance [12,13,14]. Several other studies found that the vibrotactile feedback system could be used to improve postural sway [15,16]. However, these studies did not show the effect of sensory training on balance ability, nor did they clarify the supportive relationship between sensation and balance ability.

In addition to the soles of the feet, standing balance is also significantly affected by the movement of the center of gravity, which is present in the trunk (in the region of the pelvis) when standing [17]. For this reason, a number of studies have focused on the effects of trunk function on sitting and standing balance [18,19,20]. Just as foot sole sensation is an important element in standing balance, it may also be conjectured that the tactile sensation of the trunk would be important in standing balance. However, virtually no studies of the effects of tactile sensation of the trunk on standing balance have been performed.

Balance training is used as a means to prevent falls in older adults, and this is mainly an exercise intervention. A variety of intervention programs have been developed for muscle strength training, mainly focusing on the legs and the trunk, postural balance training, and gait training [21,22,23]. As such, the most common approach to preventing falls is to maintain or improve balance performance through motor function training. A few studies have found that multisensory (visual, vestibular, and somatosensory) exercise is effective at improving balance in individuals with balance disorders [24,25]. However, studies of methods to improve balance performance through training of the tactile sensory function aspect, an important element in maintaining standing balance performance, remain rare.

While vibrotactile stimulation has been explored in several studies, these approaches have predominantly utilized vibrotactile biofeedback systems [26,27] or whole-body vibration platforms [28], rather than targeted sensory training protocols designed to enhance proprioceptive and tactile acuity. For instance, Sienko et al. [26] employed vibrotactile feedback devices as balance aids but did not seek to improve the underlying sensory processing capabilities. Furthermore, previous sensory interventions have typically concentrated on the lower extremities alone [29], overlooking the essential role of trunk somatosensation in maintaining postural stability [30]. Although somatosensory information is important for postural control, existing interventions have largely overlooked the potential for targeted sensory training, instead favoring compensatory approaches or general exercises that do not specifically address the decline in sensory acuity among older adults [26,27,28].

The present study therefore aims to reveal the characteristics of vibratory sensation function in the regions of the trunk and sole in older people using a vibration device developed by our research group, and to verify the effects of trunk and sole tactile sensation training on dynamic standing balance performance in older people. Using our specialized vibration device, we implemented a targeted training protocol that provides precise vibratory stimulation to enhance sensitivity in these key sensory regions. Our protocol possesses the potential to bridge a gap in sensory training by targeting the trunk and soles—key areas for postural control that provide vital sensory input. These findings may assess whether targeted sensory training can improve acuity and balance in older adults, offering a novel fall prevention approach.

## 2. Materials and Methods

### 2.1. Participants

The participants comprised 18 right-handed/footed older adults (nine men, and nine women) divided randomly into three groups: the control group, and the sole and trunk training groups. General information, including age and history of falls, was collected for each group. The control, trunk, and sole groups comprised eight participants (three men, and five women, mean age: 66.6 ± 3.4, mean height: 158.8 ± 7.0 cm, and mean weight: 57.2 ± 12.5 kg), five participants (five men, mean age: 71.0 ± 1.9, mean height: 169.0 ± 2.8 cm, and mean weight: 59.2 ± 4.7 kg), and five participants (one man, and four women, mean age: 66.4 ± 3.6, mean height: 157.9 ± 5.5 cm, and mean weight: 54.6 ± 4.1 kg), respectively. All participants were independent in their activities of daily livings (ALDs). In addition, participants had no history of falls within the year prior to this study. Participants were randomly divided into three groups.

To evaluate the effects of vibratory sensation training in the trunk and sole regions on standing balance, a training period (10 weeks) was established for both training groups, and vibratory sensation and standing balance were evaluated in the same manner as in the initial evaluation.

All participants received verbal and written explanations of the study before providing their written informed consent, with an assurance that their participation would be strictly voluntary, that their non-participation would not result in any disadvantage, and that their personal information would be protected. This study was conducted with the approval of the institutional review board of The University of Tokyo (approval review no. 21-230, 21-231).

### 2.2. Details of the Trunk and Sole Training

The training period for both the trunk training group and the sole training group lasted 10 weeks. Three evaluations—initial, intermediate, and final—were conducted in the first week, in the middle of the fifth week, and finally in the tenth week. Trunk training groups were carried out by delivering vibratory stimulation to the back of the trunk to train sensation to vibratory stimulation. Training was performed using two vibratory sensation test tuning forks (128 Hz). Using these tuning forks, a researcher randomly applied vibratory stimulation to one or two of the twenty sites, each with an area of 5 × 5 cm^2^, and arranged five horizontally by four vertically on the trunk region of the participant, and the participant responded when they felt the stimulation. According to the sole training, using two tuning forks, two sets of training were performed three times each on the five toes, the forefoot area from the first to the fifth toe, and the heel area. Using these tuning forks, a researcher randomly applied vibratory stimulation to one or two of the three areas, and the participant responded when they felt the stimulation. The trunk and sole trainings were carried out for 15 min at a time, three or more times a week.

### 2.3. Evaluation of Trunk and Sole Region Vibratory Sensation

**Figure 1:**
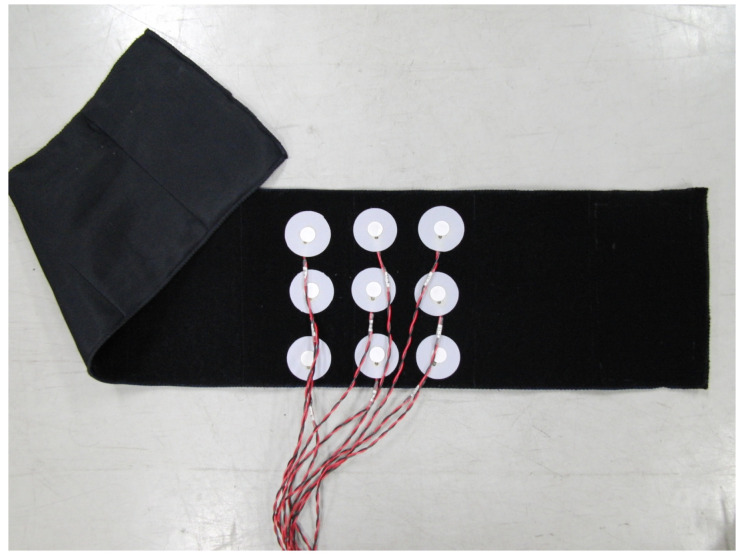
Image showing the placement of the trunk region vibrators in the belt.

**Figure 2:**
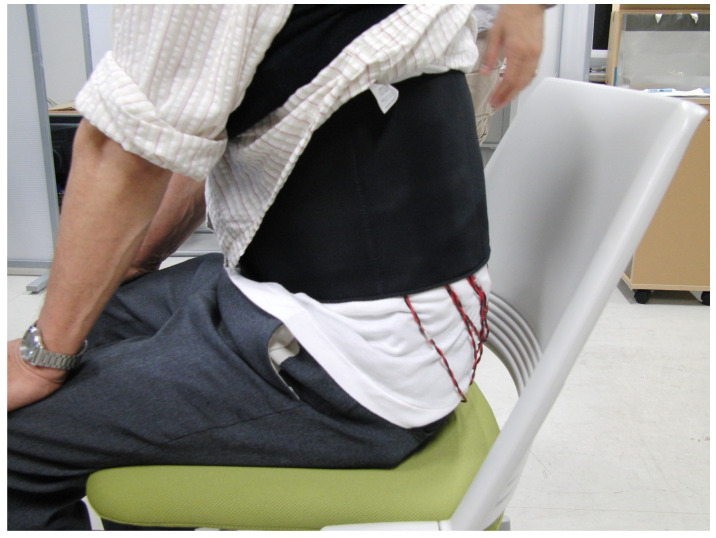
Image showing the experimental posture of sensory evaluation in the trunk region.

Vibratory sensations in the trunk and sole regions were evaluated in the three groups using the developed vibratory stimulation device (Kyowa Electronic Instruments Co., Ltd., Tokyo, Japan). The main specifications of the vibrators (diameter 14 mm, thickness 3.7 mm, weight 1.6 g) included a frequency range of 150 Hz, a voltage of 3 V, and a duration of one simulation time of 1 s. The vibratory stimulation intensity was maintained at a constant level with no variation. The vibratory sensation in the trunk region was evaluated using a developed vibratory stimulation device. Vibratory stimulation was delivered to the trunk region via a belt worn around the participant’s trunk, which was fitted with nine vibrators arranged in a pattern 15 cm vertically × 20 cm horizontally (Figure 1 and Figure 2). Participants were instructed to respond to how many vibrators they could identify. Table 1 lists the trunk vibration stimulation patterns. For a single trial, the participants were presented with 26 vibratory stimuli, and one trial each was carried out on the left, center, and right sides of the trunk, yielding a total of 78 stimuli. Three vibration patterns were presented randomly: stimulation at zero sites, stimulation at one site, and simultaneous stimulation at two sites. Additionally, for simultaneous stimulation at the two sites, the two vibrators were placed vertically, horizontally, and diagonally (Table 1).

**Figure 3:**
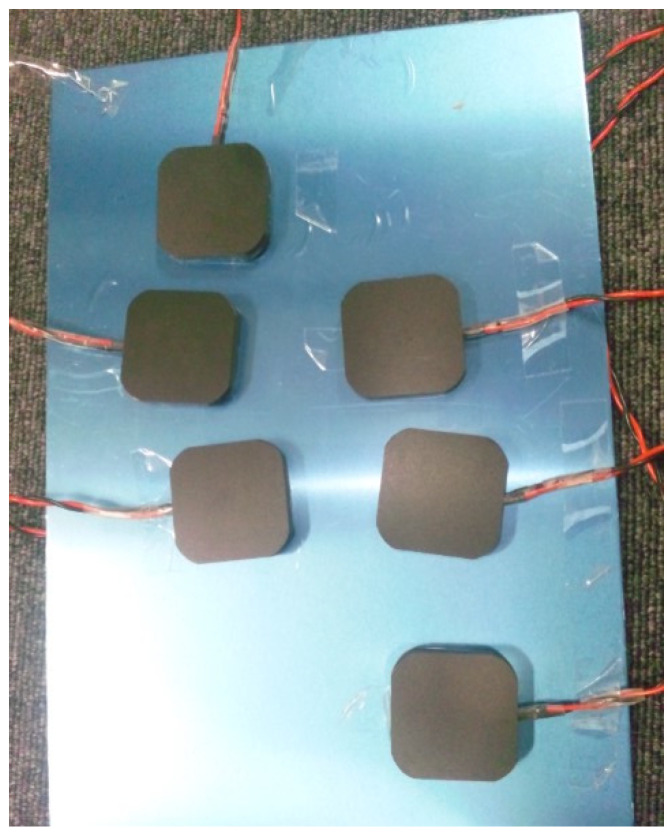
Image showing the placement of the sole region vibrators on a foot.

**Figure 4:**
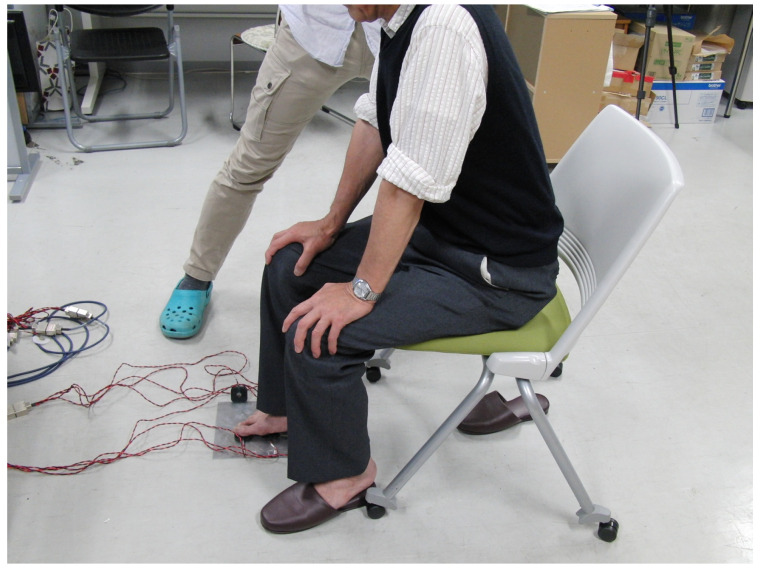
Image showing the experimental posture of the sensory evaluation in the sole region.

The conditions for the sensory test using vibration stimulation of the sole of the foot are presented in Figure 3 and Figure 4 and Table 2. Overall, six vibrators were placed on the sole vibration plate, with five one-point stimulations, seven two-point stimulations, and one without stimulation (Table 2). Under these conditions, vibration stimulation was randomly presented to the participants 15 times per trial. Stimulation was applied 45 times total across three trials.

In both the trunk and sole sensory evaluations, participants were instructed to answer whether the stimulation was given in “zero sites”, “one site”, or “two sites”. To avoid any visual or auditory influences, the participants were seated and wore eye masks and headphones for evaluation.

The vibrators used in the vibratory stimulation device were small vibration motors (Kyowa Electronic Instruments Co., Ltd.), commonly used in mobile telephones. The voltage of the vibrator used in this study was equal to or less than 3 V. The vibrator device did not directly touch the participants’ skin. The system used a one-chip microcomputer (PIC16F84A; Microchip Technology Inc., Chandler, AZ, USA) to control all vibrators. The system could produce mechanical vibrations of up to 100 Hz. The time required for the stimulus to travel to all the points was limited to 1.0 s for all patterns.

### 2.4. Evaluation of Standing Balance Using a Force Plate

**Figure 5:**
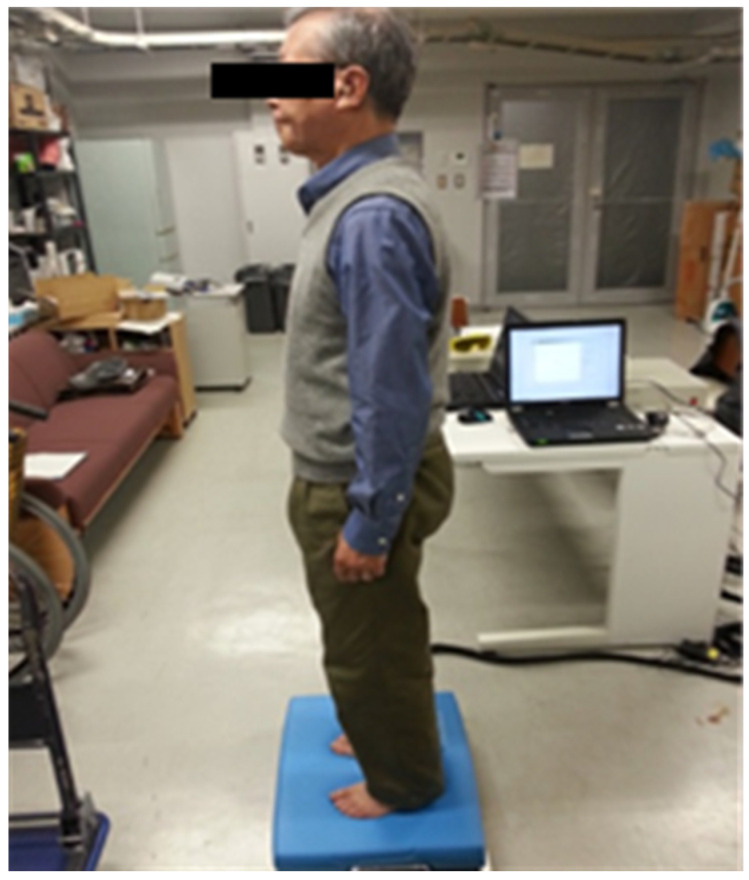
Image showing the evaluation scene of balance performance.

The measurement device for the standing balance was a force plate (Kyowa Electronic Instruments Co., Ltd.) that measured body sway parameters (Figure 5), which contained four transducers, one in each corner of the force plate, to measure the vertical forces indicating postural sway. The signals from the transducers were amplified and digitized using a computer. The computer program provided several indices of body sway calculated from the center of pressure. The accuracy and hysteresis of the force plate were ±1% and ±0.1%, respectively. The sampling rate of the force plate was 100 Hz.

To evaluate dynamic balance, the limits of stability (LoS) in the anterior, posterior, leftward, and rightward directions were measured [31]. The LoS test assessed the maximum displacement of the COP in both anteroposterior and lateral directions that participants could voluntarily maintain without altering their foot position and falling while standing upright with their knees extended. The participants performed this test standing barefoot with their arms at the sides of their bodies and eyes open, while looking at a marker placed 1.2 m in front of them. The standing position of all participants was measured barefoot under two conditions: on the force plate and on the force plate with a mattress spread over it. This method measures the sway of the body sway during standing posture where the sensation of the soles of the feet is disturbed by a mattress with maintaining the initial ankle joint position [32,33]. The study utilized a mattress (Balance pad, AIREX^®^, size: 410 × 500 × 60 mm, weight: 700 g, polyvinyl chloride resin foam) for the clinical assessment of the effect of sole tactile sensory interaction on postural stability, related to the CTSIB (Clinical Test of Sensory Interaction in Balance) [34]. The mattress provided a stimulus to the participant’s sole without the movement of the ankle joint [32].

### 2.5. Analysis

The items for analysis from the trunk and sole sensory tests included the responses to stimulation of one and two sites. The items for statistical analysis obtained from the force plate included the data from four directions in the LoS test. The total LoS test value was also calculated based on the results of the left–right and right–anterior sways.

First, analysis of variance (ANOVA) was employed to identify significant differences between responses to stimulation of one and two sites, and to examine the main effects of the training group (control, trunk, and sole groups), number of rounds (1–3; Initial, intermediate, and final evaluations), positions to vibrate, and the interactions among these factors for these responses by the number of stimulated sites (0–2). Before the ANOVA, an aligned rank transformation (ART) [35,36] was performed on the scales, as the responses were non-normally distributed. The significance of the main effects was determined using post hoc multiple comparison methods, based on the least-squares means and Tukey’s multiplicity adjustment [37,38]. Subsequently, we calculated the effect sizes, including the partial *η*^2^ and Cohen’s d, and determined the degree based on Cohen’s effect size indices [39]. The level of significance was set at 5%.

Moreover, a robust multiple regression was performed with the dynamic sway data (five values from the LoS test: value for each of the four directions, including the anterior, posterior, left, and right values, and total value for the four directions) as dependent variables, and the experimental conditions and the rate of correct responses in sensory tests as the independent variables. We also calculated Pearson’s correlation coefficients, and we checked the significance of these coefficients. The significance levels were set at 5% and 10%, respectively.

## 3. Results

### 3.1. Vibratory Sensation Evaluation

**Figure 6:**
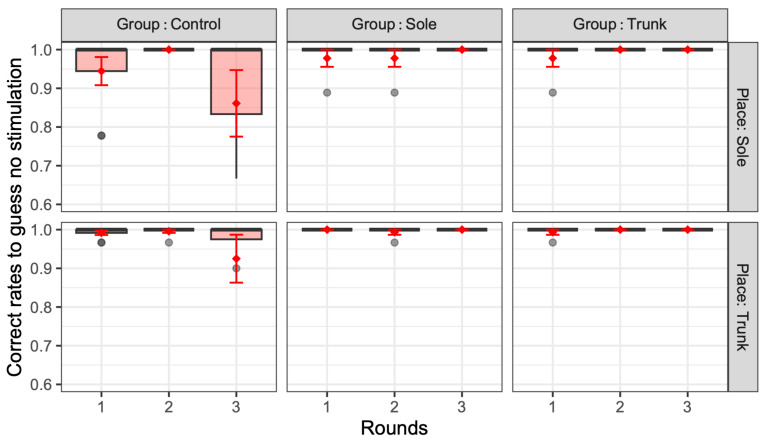
Correct rates for guessing no stimulation when there was no presented vibration. The thick black horizontal lines of these figures represent the median, while the red diamonds and error bars indicate the mean and standard error, respectively.

**Figure 7:**
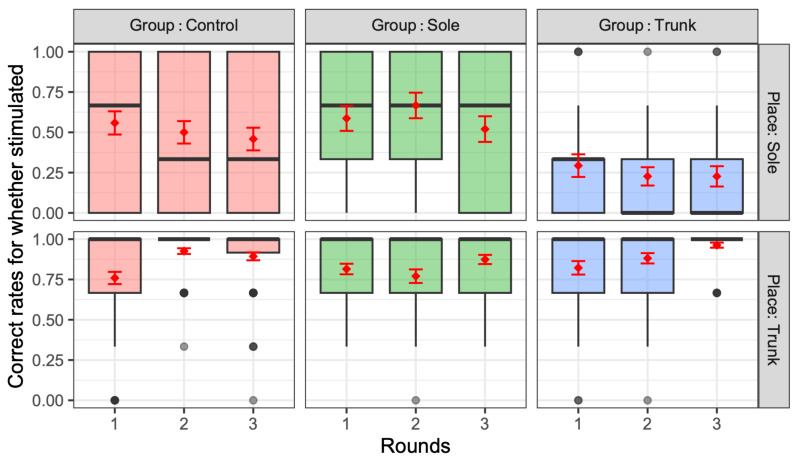
Correct rates for guessing a single vibration when a single vibration was presented to the participant. The thick black horizontal lines of these figures represent the median, while the red diamonds and error bars indicate the mean and standard error, respectively.

**Figure 8:**
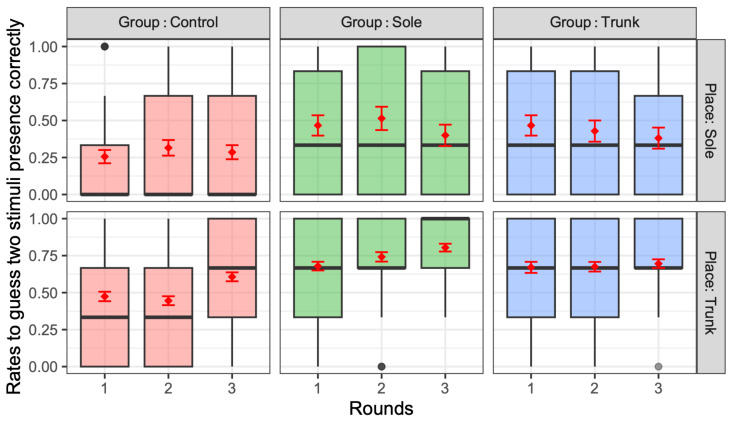
Correct rates for guessing two vibrations when two vibrations were presented to the sole and trunk. The thick black horizontal lines of these figures represent the median, while the red diamonds and error bars indicate the mean and standard error, respectively.

Figure 6, Figure 7 and Figure 8 present the correct rates for guessing no stimulation, identifying when one vibration was presented, and identifying when two stimuli were presented, respectively. In these figures, the correct rate of vibration is plotted for each training group (control, sole, and trunk) and presentation location (sole and trunk) in relation to the number of rounds (one, two, and three). The thick black horizontal lines of these figures represent the median, while the red diamonds and error bars indicate the mean and standard error, respectively. In the absence of vibration, the mean and median percentages of correct responses were generally above 90%, regardless of the group allocation, stimulus location, or number of rounds, suggesting that few participants in the experiment experienced daily phantom vibrations. However, as the number of rounds increased, the percentage of such correct responses in the sole and trunk training groups increased, while in the third round, the percentage of correct responses in the sole and trunk presentations was 100% for both the sole and trunk groups, indicating that only the control group had a significant number of errors (χ^2^(2) = 7.26, *p* = 0.027 < 0.05. The adjusted Pearson’s residual value for the control group were as follows: 2.69, *p* = 0.043 < 0.05).

Regarding the one-stimulus condition, an ANOVA performed on the correct rates confirmed a significant main effect of the training group and vibration location (*p* < 0.05). The exact statistical value of these significant factors in the correct rates was as follows: training group: *F*(1,2) = 15.8, *p* < 0.001, partial *η*^2^ = 0.04 (medium); vibration places: *F*(1,1) = 270.8, *p* < 0.001, partial *η*^2^ = 0.27 (large). Furthermore, the interactions among these factors were significant, with the exact results as follows: training group and vibration place: *F*(1,2) = 31.4, *p* < 0.001, partial *η*^2^ = 0.08 (medium). Although the significance level was not achieved, the following results are also noted: vibration place and number of rounds: *F*(1,2) = 2.83, *p* = 0.060, partial *η*^2^ = 0.008 (negligible).

The correct rates for stimulation with one vibration was lower in the trunk training group than in the other groups, especially for the presentation of vibration to the sole (t(738) = 5.0, *p* < 0.0001, d = 0.51 (medium)), while the correct rate of vibration to the trunk increased significantly with the number of rounds (r = 0.27, t(133) = 3.14, *p* = 0.002). However, there was no significant difference between the sole and trunk training groups, even when the vibration was presented to the trunk.

Regarding the correct rates of guessing the presence of two stimuli, ANOVA identified significant main effects for the training group (*F*(1,2) = 29.1, *p* < 0.001, partial *η*^2^ = 0.048, middle effect size) and vibration combinations (*F*(1,9) = 35.4, *p* < 0.001, partial *η*^2^ = 0.22, large effect size). For the number of rounds, although the *p*-value approached but did not reach the conventional significance threshold (*F*(1,2) = 2.39, *p* = 0.09, partial *η*^2^ = 0.004), the effect size suggests this factor had minimal practical impact on the results. Further, the interactions among these factors were significant, as follows: training group and vibration combinations: *F*(1,18) = 2.0, *p* = 0.008, partial *η*^2^ = 0.03 (medium); vibration combinations and number of rounds: *F*(1,18) = 1.88, *p* = 0.014 < 0.05, partial *η*^2^ = 0.028 (small).

**Figure 9:**
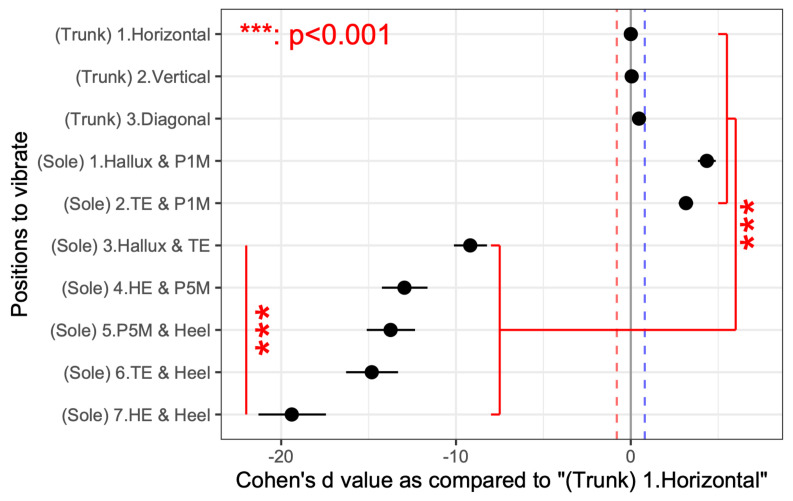
Effect size of the correct response rate for each of the two stimulus locations on the trunk and sole for participants to correctly guess the presence of the two stimuli. Effect sizes are relative to the (Trunk) 1. Horizontal condition. Significant differences were obtained through multiple comparisons. The red and blue dotted lines indicate values with large effect sizes.

**Figure 10:**
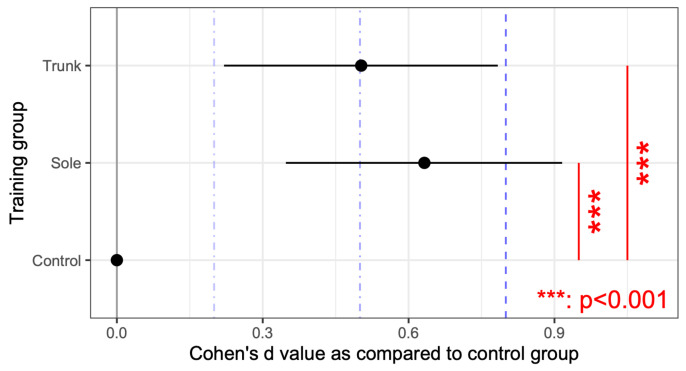
Effect size of the correct response rate for each of the two stimulus locations on the trunk and sole for participants to correctly guess the presence of the two stimuli. Effect sizes are relative to the control group. Significant differences were obtained by multiple comparisons. The blue dotted lines indicate values for which the effect size is about large (0.8), medium (0.5), or small (0.2).

**Figure 11:**
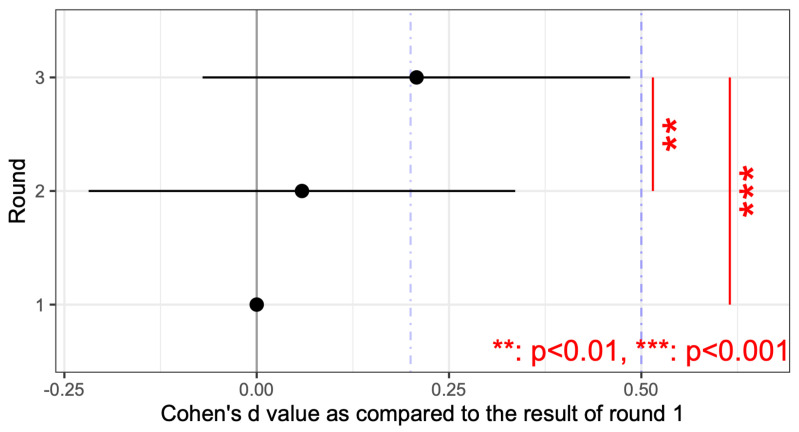
Effect size of the correct response rate for each of the two stimulus locations on the trunk and sole for participants to correctly guess the presence of the two stimuli. Effect sizes are relative to the results of round 1. Significant differences were obtained by multiple comparisons. The blue dotted lines indicate values for which the effect size is about medium (0.5), or small (0.2).

The rate of correctly guessing the presence of the two stimuli tended to be higher in both the trunk and sole training groups compared with the control group (*p* < 0.001). Moreover, the correct rates changed with repetition, with the training group showing a significant increase in the correct rates for trunk vibrations. Figure 9 presents the correct rates for each combination of the two stimulation points in the sole and trunk, with an effect size d, as compared with the performance of the horizontal vibration on the trunk for convenience. For the sole, the percentage of correct responses was significantly higher when stimulation was applied to the proximal first metatarsal (P1M) and anywhere else vs. when stimulating the other two points of the sole. In particular, the correct response rate was significantly lower when the heel was stimulated than when the first proximal metatarsal was stimulated. The two-point discrimination results for the trunk were also significantly more accurate than those for the sole, except when the proximal first metatarsal and another point were stimulated. For convenience, Figure 10 presents the relative correct rates when two vibrations were presented on the trunk for each training group, with an effect size d, when compared to the control group. The correct response rates were significantly higher in the trunk and sole training groups than in the control group. Figure 11 presents the correct rates in the training sessions for convenience, with an effect size d, when compared to the results of round 1. The correct rates for round 3 were significantly higher than those for rounds 1 and 2.

### 3.2. Evaluation of Standing Balance Performance with a Force Plate

**Figure 12:**
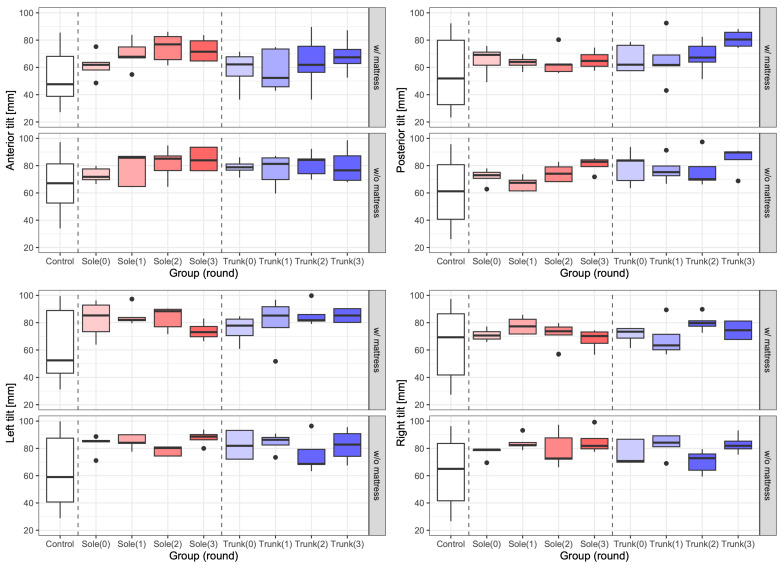
Dynamic balance performance with and without a mattress. Top left: anterior tilt, top right: posterior tilt, bottom left: left tilt, bottom right: right tilt. The top and bottom of each graph indicate the case with and without a mattress, respectively. Round 0 refers to the period before the intervention. The box plots in white, red and blue are the results for the control, sole training and trunk training groups, respectively.

**Figure 13:**
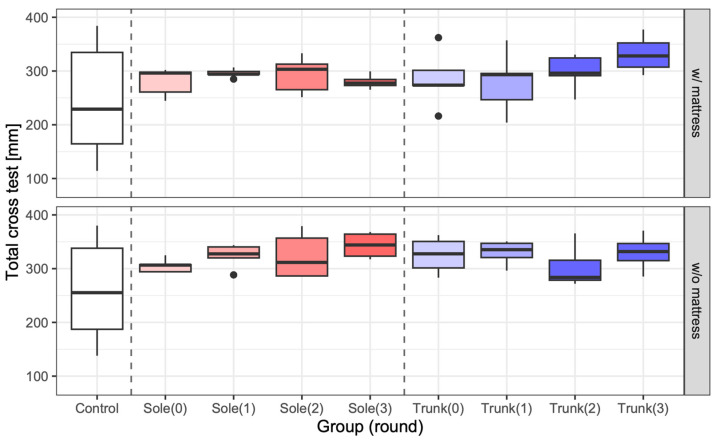
Performance of the total LoS test with and without a mattress. Round 0 refers to the period before the intervention. The box plots in white, red and blue are the results for the control, sole training and trunk training groups, respectively.

Dynamic balance was evaluated using the mean maximum displacement values with the body inclined in the anterior, posterior, right and left directions (tilts) while standing. Figure 12 shows the tilt performance difference in the training groups from the first to fourth rounds and control groups in these directions with or without a mattress. Figure 13 shows the results of the total LoS test for the same groups with and without a mattress. In the initial evaluation, the maximum displacements in the control group were approximately 60 mm anterior tilt, 54 mm posterior tilt, 60 mm right tilt, and 57 mm left tilt. Conversely, the maximum displacement values in the trunk-training group were approximately 79 mm of anterior tilt, 78 mm of posterior tilt, 74 mm of right tilt, and 88 mm of left tilt. In the post-training evaluation, the maximum displacement values in the trunk-training group were approximately 80 mm anterior tilt, 85 mm posterior tilt, 83 mm right tilt, and 82 mm left tilt.

Table 3 presents the statistical results of the effects of the experimental conditions on dynamic balance performance. In general, significant differences were found in some balance metrics in the trunk and sole training groups against the control group. While significant differences were confirmed in the number of rounds and the use of the mattress in the anterior and posterior tilts as well as in the total tilt of LoS test, no significant differences in the number of rounds were confirmed in the left and right tilts. Furthermore, with regard to the correct response rate for vibration, the higher the discriminative ability in the trunk, the greater the tilts, but there was no significant relationship between the correct response rate for the sole and the tilts. Table 4 shows the correlation coefficient between correct rates of vibrations and LoS tilts. When the correct response rate was high for the two vibrations on the sole and trunk, a correlation was confirmed in which the tilt value became significantly larger. According to the value of the correlation coefficient, while the correct response rate of the two vibrations on the sole was significantly correlated, that of a single vibration on the sole would correlate negatively to the tilt values. However, as no significant difference was found in the correct rates for the sole in Table 3, the significant correlation on the sole might be less dominant than the sole training group itself.

Therefore, based on the results described in Figure 12 and Figure 13, as well as Table 3 and Table 4, vibration training for both the trunk and the sole positively impacts balance ability. The larger sway seen in the training groups versus controls reflects a better capability to shift the center of pressure towards stability limits while maintaining balance. This increased movement range signifies improved dynamic postural control rather than instability, as supported by previous research [31]. In dynamic balance tasks such as our LoS assessment, larger controlled movements usually suggest superior balance performance and increased movement confidence, contrary to static balance tasks, where reduced sway is generally preferred [40]. However, when examining the relationship between discrimination ability and balance ability, it was found that while vibration discrimination ability for the trunk could have a positive relationship with balance ability, the discrimination ability for the soles may not affect to balance ability.

When comparing the training groups, the anterior and posterior tilts and the total LoS test for both the trunk and sole training groups were larger than those of the control group for factors where significant differences were observed. In the anterior tilt, the sole exercise group showed significantly greater improvement than the trunk exercise group (*p* = 0.013 < 0.05, d = 0.27 (small)), whereas in the posterior tilt, the trunk exercise group showed significantly greater improvement than the sole exercise group (*p* < 0.001, d = 0.46 (medium)). There was no significant difference in the left and right tilts between the plantar and trunk groups (*p* > 0.10, d < 0.2 (negligible)).

Thus, the results indicated that after 10 weeks of training, there could be a significant improvement in dynamic balance performance in the sole and trunk training groups compared with the control group.

## 4. Discussion

The flexibility and stability of the foot arches are closely related to standing and walking balance [41]. In particular, a low medial longitudinal arch (MLA) can negatively affect balance by disrupting foot stability and the relationship between the foot and the floor [42,43,44]. Moreover, several studies have reported a relationship between arch pathologies and poor postural sway in young adults and seniors aged over 65 years [45]. Because the longitudinal arch is known to have a significant influence on balance, in the present study, we decided to present vibration stimulation methods focusing on the medial arch of the sole, which is particularly important.

Several researchers have proven the effectiveness of vibrator stimulation at improving dynamic balance [12,46,47]. For the plantar vibrator stimulation, Khalifeloo et al. [48] indicated that vibratory stimuli applied to the plantar region of patients’ post-stroke could have beneficial effects on dynamic balance, as assessed by the timed “Up & Go” (TUG) test [49,50]. Önala et al. further showed the effectiveness of local vibration stimulation training applied to the plantar region of the foot with conventional rehabilitation on static and dynamic balance in stroke patients [51]. The results of the present study showed that the effects of 10 weeks of plantar vibration stimulation training may improve dynamic balance ability.

Regarding the influence of trunk vibration somatosensory stimulation on standing balance, Goldberg et al. reported that trunk repositioning errors (TREs) were reliable and valid measures of underlying balance impairment in older adults, indicating that TREs may ultimately be proven to be a marker of fall risk. Further, TREs indicated the assessment of trunk position sense difficulty [52]. In another study, Sonasath et al. reported that children with spastic diplegic C.P. with better lumbar position sense scored greater in static and dynamic balance [53]. To the best of our knowledge, studies investigating the effects of somatosensory stimulation on the trunk on balance are rare. In this study, similar to plantar vibration stimulation training, the results showed that trunk vibration stimulation training could be expected to have an effect on dynamic balance ability.

Overall, this study found no significant difference between the two groups in the vibration sensation training group for the sole and the trunk (Figure 10); however, the correct response rate with stimulation of the two sites was generally lower than that with stimulation of one site after training (Figure 7 and Figure 8). This is similar to the finding that older adults tend to show decreased sensitivity to vibratory stimulation in threshold tests for vibratory stimulation delivered to their legs [12,14,54]. On the other hand, the correct response rate for vibration stimulation showed a significant increase as the number of training sessions increased (Figure 11). Therefore, it was suggested that vibration sensation training enhanced vibration sensation. Although no significant difference was observed in this study, the fact that the effect size was larger when the vibration stimulators were positioned diagonally than when they were arranged horizontally or vertically (Figure 9) is consistent with the results of previous studies that investigated tactile pressure sensation in the fingers [55,56].

According to the results of Table 3 and Table 4, there was a significant positive correlation between the correct response rate for each of the two-point stimuli on the trunk and sole, and the limits of stability (LoS) test. However, for the sole, the positive response rate for single-point stimulation was negatively correlated with the ability to maintain dynamic postural stability. In other words, improving sensation through sensory training of the trunk and sole could be related to improving dynamic balance. However, improving sensation at a single point on the sole may have a greater effect on static dynamic balance than dynamic balance [6,11,15,56]. A one-point stimulus is a local stimulus and a two-point stimulus indicates the direction of the stimulus, providing a sense of spatial awareness related to dynamic balance. Further investigation is needed to explore how single- and two-point stimulations affect static and dynamic standing balances. These findings suggest that improving the tactile function of the trunk and the sole is important for posture control. Additionally, the results in Table 3 and Table 4 also highlight the importance of discriminating between multiple vibrations. These findings suggest that improving the tactile function of the trunk and the sole is important for posture control, and the results in Table 4 also suggest that discriminating between multiple vibrations is particularly essential. Indeed, it has been reported that the simultaneous stimulation of two sites requires more complex processing than a single stimulation of one site, and hence, it is more difficult for older people to perceive [57,58].

Mechanoreceptors that react to the simultaneous stimulation of the two sites are likely to affect spatial resolution and postural changes. Improvement in sensing and reaction to stimulation of the two sites may, therefore, allow a person to perceive movements of the center of gravity and thus control posture, meaning that trunk sensory training may be important for postural control.

The results further indicated that plantar stimulation improved the dynamic balance ability in the direction of the anterior and posterior body sway. Parsons et al. [59] further indicated that plantar sensation on the affected side of patients with stroke was correlated with the Berg Balance Scale (BBS) [60] and the anteroposterior center of pressure variability in the eyes-closed standing position. Moreover, Wanderley et al. reported that the application of local vibration to the plantar region has beneficial effects on postural control along the anteroposterior axis [61]. These findings are consistent with our results.

In the present study, the results of the logistic regression analysis showed an association between the correct response rate with vertical stimulation of the two sites and the forward and backward displacement values in the LoS test; however, equations were only found for the total anteroposterior movement of the center of gravity, which is easy to understand as the movement of the trunk, and vertically paired stimulation of the trunk region, which had a good rate of correct responses. Several prior studies have reported that tactile sensations, such as touch–pressure sensation and vibratory sensations of the sole, are important in relation to standing balance and gait performance [62,63,64]. Several studies have also reported that the reactions to tactile sensation of the sole are dominant over reactions to visual and vestibular sensations as reactions for postural control [65,66]. From this, it could be concluded that tactile sensations in the soles and trunk are particularly important for avoiding falls while standing or walking. The high correlation between the tactile sensation of the trunk and the dynamic center of gravity data in the present study indicates the importance of sole and trunk sensory in preventing falls.

Several scholarly articles address the physiological mechanisms through which vibration sensory stimulation training influences human physical movement. Vibratory sensory stimulation impacts motor control by activating sensory receptors and integrating information within the somatosensory and association cortices. This study posits that vibration applied to the trunk or soles may enhance postural muscle activity in older adults experiencing age-related sensory decline; however, the underlying neural mechanisms require further investigation [67]. This study examined whether subsensory vibratory noise generated by a piezoelectric insole could enhance sensation, balance, and gait among older individuals. The findings indicated improvements in balance and gait, suggesting that applying stochastic resonance to the foot’s sensory systems may contribute to fall prevention. In contrast to our study, previous research employed short-term stimulation (lasting less than two weeks), specifically targeting only the medial plantar arch [68]. Neck muscle vibration (NMV) and trunk proprioception significantly influence body perception and postural control. NMV induces an illusion of muscle stretching, thereby shifting the center of pressure (COP), while trunk vibration modifies gait trajectory. Both observations imply that vibratory stimulation can effectively modulate COP, showcasing its utility as a tool for postural training [69]. Sensory training aimed at both the trunk and soles can enhance standing balance, although their individual contributions remain ambiguous. Further research should investigate the effects of trunk and plantar sensations on postural control across various movements, such as standing, walking, and different phases of gait, to optimize sensory training tailored to each posture.

In clinical implications, vibratory stimulation is inherently safe, poses no risk of side effects, and is cost-effective, making it highly practical. The results of this study have clarified that sensory training of the soles of the feet or the trunk is connected to improved dynamic standing balance ability. Accordingly, when the standing balance ability of older adults is decreased or lost, we believe that it is important to actively evaluate and train the senses of the trunk and the soles of the feet, in addition to the conventional balance training practiced in clinical settings. Furthermore, the type of shoe insole and trunk orthosis with vibratory stimulators are considered valuable as portable sensory training devices that can facilitate the sensation of the soles of the feet and the trunk. By utilizing a vibration device or a balance training app based on IMU (Inertial measurement unit), motion analysis can be conducted in a game-like manner, which can boost user motivation and create a more effective training method under appropriate medical professional guidance.

### Limitations

Although the LoS data were used as an index of dynamic balance ability in this study, adjustments may be necessary when comparing groups of different heights. To further clarify the relationship between dynamic balance ability and sensory training, it is essential to measure trunk and lower extremity joint movements and muscle activity using various motion analysis methods to analyze the relationship factors with higher accuracy. Additionally, it is important to consider measuring brain activity to support the impact of sensory training on standing balance ability. When collecting data on older adults’ characteristics, it is essential to acknowledge that differences in age, slight variations in sensory thresholds, and personal physical activity abilities may also influence responses to vibratory stimulation. This will include examination of the superiority of the effects on standing balance of a trunk training group and a sole training group, examination of the effects on standing balance of long-term sensory training of 12–14 weeks or more with a larger sample size, examination of sex differences, and examination of the specifications of the vibratory device, including miniaturization and vibratory stimulation at different frequencies.

The sample size in this study may have been insufficient. Concerning the result of the *χ*^2^ test presented in Section 3.1, which indicated that the control group committed significantly more errors than both the sole and trunk training groups as the number of rounds increased, the power analysis conducted using the *pwr* (ver. 1.3.0) and *pwrss* (ver. 0.3.1) packages in R [70,71] revealed a value of 0.67, which falls short of the threshold of 0.8. However, a post hoc power analysis of the ANOVA results presented in the same section, conducted using the *Superpower* package (ver. 0.2.0) of the R software (ver. 4.2.2) [72], indicated that the statistical power exceeded 0.8 for all the factors and interactions that demonstrated significance. When we analyzed the post hoc power of the significant results from the multiple comparisons using the *PMCMRplus* package (ver. 1.9.10) in R software [73], we found that the power exceeded 0.8 whenever a significant difference was observed. Furthermore, concerning the outcomes of the robust regression illustrated in Figure 3, the power analysis based on *R*^2^ utilizing the aforementioned *pwr* and *pwrss* packages [70,71] revealed that all tilt parameters exhibited a power greater than 0.8. This observation also indicates that internal validity was upheld to some extent, despite the limitations imposed by the sample size. Using the same power analysis packages, we also conducted a post hoc power analysis on the correlations with a medium effect size presented in Table 4 and found that the statistical power exceeded 0.8, even for the small samples in this study. In instances where a small correlation coefficient is observed, if the absolute value does not surpass 0.25, the statistical power remains below 0.8. Nonetheless, despite the significance and sufficient statistical power yielded by these results, limitations persist regarding the demonstration of consistent internal validity and the establishment of a clear causal relationship. Therefore, additional studies are imperative to thoroughly investigate both internal and external validities, as well as precise causality, involving a larger participant pool. At that time, it will be crucial to examine the various dynamic and static balance metrics in relation to diverse participant characteristics, such as age, gender, and personality traits, within an expanded group of older adults encompassing a range of influencing factors.

## 5. Conclusions

In the present study, we investigated the effects of vibratory stimulation training on standing balance using a new vibratory sensation test device. The participants were all elderly individuals aged 65 years or older, who were divided into two training groups and a control group for comparison and analysis. Trunk or sole region vibratory sensation training was carried out for 10 weeks. Balance was measured as dynamic balance, while vibratory sensation was evaluated as the rate of correct responses. Overall, the trunk and the sole training group showed an improvement in the correct response rate over time, as well as an improvement in dynamic balance performance. A significant relationship was found between vibratory sensation and dynamic balance performance, both of which are linked to the causes of falls. The results of a series of analyses indicated that the intervention in this study led to improvements in the correct response rate for vibrations and balance performance. These improvements, combined with the effects of the intervention itself, led to improvements in dynamic balance performance, indicating that they could be used as interventions to reduce falls in older adults. To validate and expand upon our findings, future work should examine the superiority of the effects of trunk and sole training on standing balance and the effects of long-term sensory training for 12–14 weeks or more on standing balance using a larger sample size.

## Figures and Tables

**Table 1:** Overview of the vibrator arrangement and vibration stimulation pattern for the trunk vibration sensation evaluation.

	Condition	Vibrators in the Trunk Position	
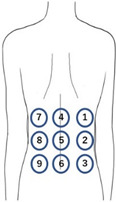	One stimulus	1, 2, 3, 4, 5, 6, 7, 8, 9	
	Two stimuli	(Vertical)	1–2, 4–5, 7–8, 2–3, 5–6, 8–9
		(Horizontal)	1–4, 4–7, 2–5, 5–8, 3–6, 6–9
		(Diagonal)	1–5, 5–9, 7–5, 5–3
	No stimulus	N/A	

**Table 2:** Overview of the vibrator arrangement and vibration stimulation pattern for plantar vibration sensation evaluation. The a–f symbols in vibrators correspond to the a–f symbols on the sole shown on the left.

	Condition	Vibrators	Plantar Position(s)	(Abbreviation)
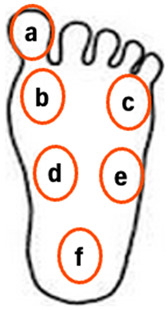	One stimulus	a	Hallux	Hallux
		b	Thenar eminence	TE
		c	Hypothenar eminence	HE
		d	Proximal first metatarsal	P1M
		f	Heel	Heel
	Two stimuli	a–b	Hallux–Thenar eminence	Hallux–TE
		a–d	Hallux–Proximal first metatarsal	Hallux–P1M
		b–d	Thenar eminence–Proximal first metatarsal	TE–P5M
		b–f	Thenar eminence–Heel	TE–Heel
		c–e	Hypothenar eminence–Proximal fifth metatarsal	HE–P5M
		c–f	Hypothenar eminence–Heel	HE–Heel
		e–f	Proximal fifth metatarsal–Heel	P5M–Heel
	No stimulus	N/A	No vibration	No vibration

**Table 3:** The *p*-values acquired by robust multiple regression analysis for rounds, mattress usage, correct rates to the two vibrations, places to vibrate, and training groups with dynamic balance performances (LoS test). The results are rounded to the fourth decimal place.

	Anterior Tilt		Posterior Tilt		Right Tilt		Left Tilt		Total Cross Test	
	*p*		*p*		*p*		*p*		*p*	
Rounds	<0.001	***	<0.001	***	0.130		0.415		0.029	*
Mattress usage	<0.001	***	<0.001	***	0.008	**	0.169		<0.001	***
Correct rates (sole)	0.626		0.324		0.896		0.599		0.550	
Correct rates (trunk)	0.024	*	0.003	**	0.004	**	0.005	**	0.015	**
Training group (sole)	<0.001	***	<0.001	***	0.007	**	0.007	***	<0.001	***
Training group (trunk)	<0.001	***	<0.001	***	0.001	**	0.001	***	<0.001	***

***: *p* < 0.001, **: *p* < 0.01, *: *p* < 0.05.

**Table 4:** The correlation coefficient between correct rates of vibrations (column) and LoS tilts (row). Orange and red bold text indicates significant coefficients (*p* < 0.05) with small and medium effect sizes by Cohen’s criterion [39].

	Sole			Trunk		
	0 Vibration	1 Vibration	2 Vibrations	0 Vibration	1 Vibration	2 Vibrations
Anterior tilt	0.10	−0.06	**0.19**	0.16	−0.02	**0.41**
Posterior tilt	0.08	**−0.29**	0.16	0.08	0.05	**0.43**
Right tilt	0.10	**−0.19**	**0.27**	0.01	0.03	**0.45**
Left tilt	0.07	**−0.20**	**0.28**	0.08	−0.02	**0.48**
Total tilt	0.10	**−0.21**	**0.25**	0.09	0.01	**0.49**

## Data Availability

The datasets analyzed during this study are available from the corresponding author upon reasonable request.
